# Evaluation of AI-Predicted GH11 Xylanase Models Against a Previously Unreported Experimental Structure: Implications for Conformational Accuracy and Ligand Binding

**DOI:** 10.3390/ijms27031370

**Published:** 2026-01-29

**Authors:** Ki Hyun Nam

**Affiliations:** College of General Education, Kookmin University, Seoul 02707, Republic of Korea; structure@kookmin.ac.kr

**Keywords:** protein structure, artificial intelligence, ESMFold, AlphaFold, RoseTTAFold, xylanase, GH11

## Abstract

Artificial intelligence (AI)-based structure prediction tools have emerged as powerful methods for understanding previously unsolved structures. AI-predicted models are widely used for protein function identification, drug development, and protein engineering. Although AI-predicted structures offer significant opportunities to advance research, their inaccuracies can lead to misinterpretations of molecular mechanisms. Thus, evaluating the structural differences between AI-predicted and experimental structures is crucial for accurately understanding molecular mechanisms and guiding the design of subsequent experiments. In this study, the previously unreported crystal structure of xylanase from *Hypocrea virens* (HviGH11) was compared with the structures predicted by ESMFold, AlphaFold2, AlphaFold3, and RoseTTAFold. The overall fold of HviGH11 was highly similar between the experimental and AI-predicted models; however, the conformation of the thumb domain of the protein varied across the models. The substrate-binding cleft of experimental HviGH11 was similar to that in the model structures generated by ESMFold, AlphaFold2, and AlphaFold3, but significantly different from those in the model structures generated by RoseTTAFold. The substrate docking study illustrated that the binding mode of xylohexaose in the substrate-binding cleft differed between the experimental and AI-predicted HviGH11 structures. These findings provide insights into the applications of AI-predicted models and offer guidance for appropriate application in structural and functional studies and biotechnology.

## 1. Introduction

The three-dimensional structures of proteins provide critical insights into their molecular mechanisms, enzymatic activities, protein–ligand interactions, and conformational dynamics [1,2]. Such structural information plays an essential role in understanding fundamental biology and the practical applications of proteins in drug discovery, protein engineering, and synthetic biology [3,4,5,6,7]. Techniques such as X-ray crystallography, serial crystallography, cryo-electron microscopy (cryo-EM), nuclear magnetic resonance spectroscopy, and micro-electron diffraction have been shown to provide reliable structural information of proteins [8,9,10,11,12]. However, the approaches for experimental protein structure determination often face technical challenges, such as difficulties in protein expression, maintaining protein solubility and stability, high-quality sample preparation, and data collection and processing [13,14]. Computational structure prediction methods have been extensively developed to address these limitations [15]. Traditional approaches, including homology modeling tools such as SWISS-MODEL [16] and MODELLER [17], infer unknown protein structures based on sequence similarity to proteins with known structures. These methods are useful for generating models, particularly when reliable templates are available; however, their accuracy is often limited in regions lacking structural homologs or in flexible domains.

The application of artificial intelligence (AI) and deep learning to protein structure prediction represents a major breakthrough in recent years [18,19]. Notably, AlphaFold2 (AF2), developed by DeepMind, achieved unprecedented accuracy, often reaching near-experimental resolution [20]. AlphaFold3 (AF3) further integrates small-molecule and nucleic acid interactions, broadening its applicability with state-of-the-art accuracy in predicting biomolecular complexes and ligand interactions [21]. RoseTTAFold (RF), developed by the Baker lab, provides accurate and fast predictions through a three-track network architecture [22]. ESMFold (ESM) is a transformer-based protein language model developed by Meta AI [23]. It predicts protein structures directly from primary sequences without requiring multiple sequence alignments. This significantly reduces computational cost and inference time, enabling rapid and large-scale structure prediction and facilitating protein design and engineering applications [23]. These AI-based tools have revolutionized the field of protein structure prediction and are widely used across biology and biotechnology research. For example, AI-predicted structural models are widely utilized as initial models for molecular replacement in X-ray crystallography or as starting models in cryo-EM structure determination [24,25,26,27]. In various biochemical studies, AI-predicted models are frequently used to analyze the functions of target proteins or mutants lacking experimentally determined structures [28,29]. Moreover, AI-predicted structures serve as valuable tools in drug discovery [30,31] and protein engineering [32,33], particularly when experimental structural data are unavailable. Therefore, AI-predicted structural models have contributed significantly to several research areas; however, they are not direct replacements for experimentally determined structures [34]. For instance, current AI models do not account for the post-translational modifications, and they cannot capture dynamic structural changes [21,35]. In terms of ligand-binding accuracy, they do not match the precision achieved by experimentally derived structures [36]. Accordingly, although AI-predicted structures are valuable for hypothesis generation, they might fail to accurately represent molecular mechanisms of action [34]. In particular, inaccurate predictions can mislead downstream applications such as protein engineering and drug development. Therefore, a critical evaluation of the structural differences between AI-predicted models and experimentally determined structures is essential, as it might inform future experimental design and contribute to the improvement of AI-based prediction tools. Meanwhile, because AI prediction tools are trained on previously solved experimental structures, they might exhibit high similarity to known structures [20,21,22,23]. Therefore, to more objectively assess the accuracy of AI-predicted structures, it is important to perform comparative analyses using previously unsolved protein structures.

In the present study, the previously unreported crystal structure of the glycoside hydrolase family 11 xylanase (HviGH11) from *Hypocrea virens*, a hemicellulolytic enzyme that plays a critical role in biomass degradation and exhibits potential applications in biofuel production and industrial biotechnology, was determined at 1.95 Å resolution. This experimentally determined HviGH11 structure was compared with the models predicted by ESM, AF2, AF3, and RF. Structural similarities and differences between the experimental and AI-predicted models were analyzed in terms of overall folding, substrate-binding clefts, and active site configuration. Ligand docking was performed on the experimental and AI-predicted model structures. These results provide valuable insights into the current capabilities and limitations of AI-based structure prediction tools and offer practical guidance for the application of these tools in structural biology and biotechnology.

## 2. Results

### 2.1. Experimental Structure of HviGH11

The HviGH11 crystal structure belonged to the orthorhombic space group P2_1_2_1_2_1_, containing one molecule in the asymmetric unit, and it was determined at a resolution of 1.95 Å (Table 1).

**Figure 1:**
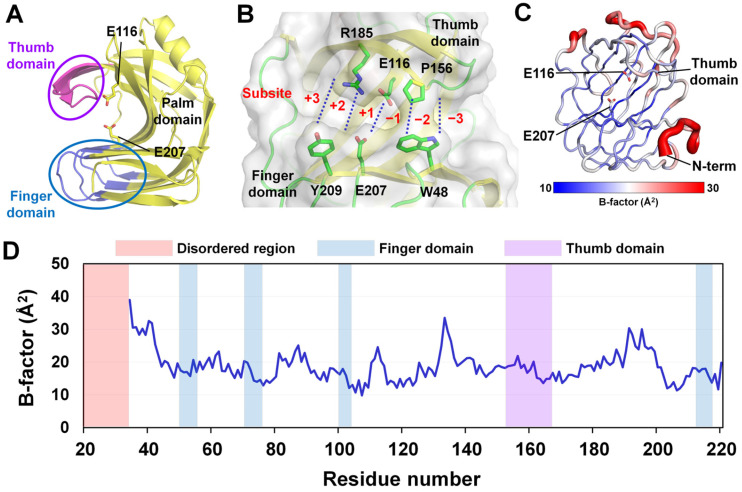
Crystal structure of HviGH11. (**A**) Cartoon representation of the HviGH11 structure, containing the palm, finger, and thumb domains. The active site is indicated by a stick. (**B**) Close-up view of the substrate-binding cleft of HviGH11, containing six subsites ranging from +3 to −3. (**C**) B-factor putty representation of the HviGH11 structure. (**D**) B-factor plot of the HviGH11 structure.

The electron density maps of the HviGH11 structure were clearly observed for model building, excluding 14 residues at the N-terminal (^19^APTESVEVEKRQTI^33^). The final model structure of HviGH11 was built from Gly34 to Ser220, indicating that the N-terminal region is flexible. The R_work_ and R_free_ values of HviGH11 were 17.00% and 20.18%, respectively. HviGH11 structure comprised one α-helix and 12 β-strands, forming the β-jelly roll fold typical of GH11 xylanases. HviGH11 sequence comprised palm, finger, and thumb domains (Figure 1A). The substrate-binding cleft was located between the thumb and finger domains and contained six subsites (−3 to +3) for substrate recognition (Figure 1B). Two catalytic glutamate residues (Glu116 and Glu207) were located near the center of the cleft between subsites −1 and +1. B-factor putty representation and B-factor analysis indicated that the N-terminus and the region opposite the thumb domain were more flexible than other areas, whereas the finger, palm, and thumb domains were relatively more rigid (Figure 1C,D).

### 2.2. AI-Predicted HviGH11 Structures

To evaluate the accuracy of AI-predicted models, structural models of HviGH11 were generated using ESM, AF3, and RF. AF3 and RF produced five structural models each, whereas ESM generated a single model by default. The AF2 model was obtained from the AlphaFold Protein Structure Database, which provides a single structural model. All AI-predicted structures of HviGH11 adopted the canonical β-jelly roll fold (Supplementary Figure S1), similar to the experimentally determined crystal structure, although differences were observed in local structural features.

**Figure 2:**
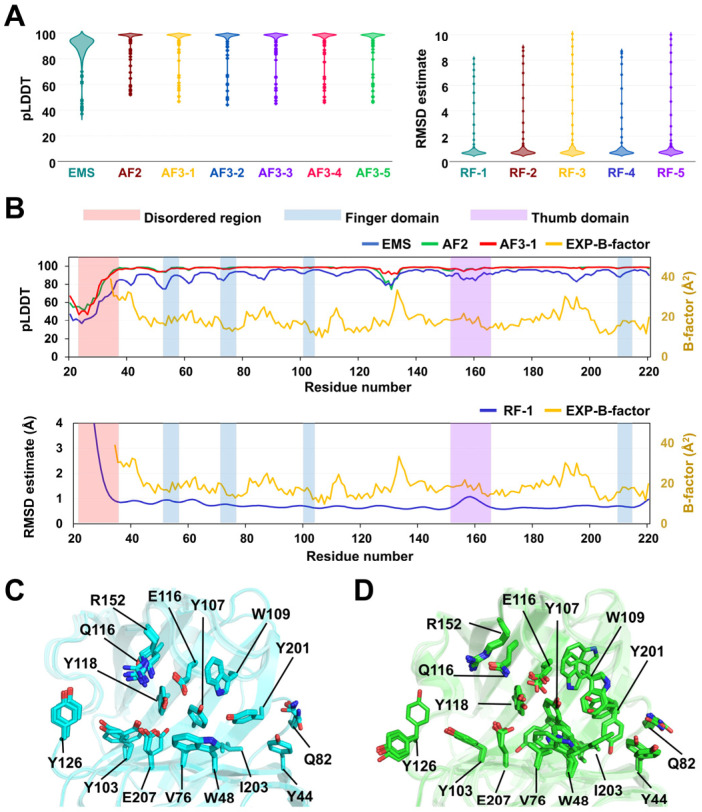
Analysis of the AI-predicted HviGH11 models. (**A**) Violin plots showing the pLDDT values predicted by ESM, AF2, and AF3 (AF3-1 to AF3-5) and the RMSD estimates predicted by RF (RF-1 to RF-5). (**B**) Confidence score plot showing per-residue pLDDT values for ESM, AF2, and AF3, and RMSD estimates for RF, overlaid with the experimental B-factor values of EXP-HviGH11. Superimposition of the five model structures of (**C**) AF3-HviGH11 and (**D**) RF-HviGH11.

The ESM-HviGH11 model displayed an average predicted Local Distance Difference Test (pLDDT) score of 88.0 with relatively low confidence in the N-terminal region (Ala20–Pro36: 0.516, Figure 2A,B). The AF2-HviGH11 model exhibited an average pLDDT of 95.25 (Figure 2A). The pLDDT plot indicated low confidence in the N-terminal region (Ala20–Arg30) with an average pLDDT of 58.48, whereas the remainder of the β-jelly roll core was predicted with high confidence (Figure 2B).

The five AF3-HviGH11 models exhibited a predicted TM (pTM) score of 0.93 and an average pLDDT value of 95.35 (Figure 2A). Structural superimposition of the five AF3 models yielded root mean square deviation (RMSD) values ranging from 0.20 to 0.65 Å, showing positional variation in the unstructured N-terminus and the thumb domain (Supplementary Figure S2). Notably, four of the five models displayed an identical conformation of the catalytic residue Glu207, whereas in one model, Glu207 was rotated toward Glu116, the other catalytic residue (Figure 2C). The shortest distances between the OE atoms of Glu116 and Glu207 in AF3 models 1–5 were 6.02, 5.79, 5.89, 5.72, and 6.12 Å, respectively. Furthermore, the side chain of Tyr103 was oriented toward the active site in two models, whereas in the other three models, it pointed toward subsite +2 (Figure 2C).

The five RF-HviGH11 models exhibited an average estimated RMSD of 1.06 Å, with particularly high predicted error scores recorded for the N-terminal region (Ala20–Arg30: ~6.38 Å, Figure 2A,B). Superposition of the five RF models revealed RMSDs ranging from 0.55 to 1.05 Å, showing positional variation in the unstructured N-terminus, the finger domain, and the thumb domain (Supplementary Figure S2). In addition, significant differences were observed in the side-chain conformations of catalytic and substrate-binding residues (Figure 2D). The conformations of the side chains of the catalytic residues Glu116 and Glu207 varied substantially among the models. The shortest distances between the OE atoms of Glu116 and Glu207 in the five RF models were 6.67, 6.07, 6.91, 7.17, and 6.33 Å, respectively. Moreover, residues involved in substrate recognition, such as Tyr44, Trp48, Gln82, Tyr107, Trp109, Tyr201, and Tyr126, exhibited substantial conformational variability (Figure 2D). These structural variations resulted in differences in the shape and electrostatic surface of the substrate-binding cleft of HviGH11 (Supplementary Figure S3).

### 2.3. Structural Comparison of Experimental and AI-Predicted HviGH11 Structures

**Figure 3:**
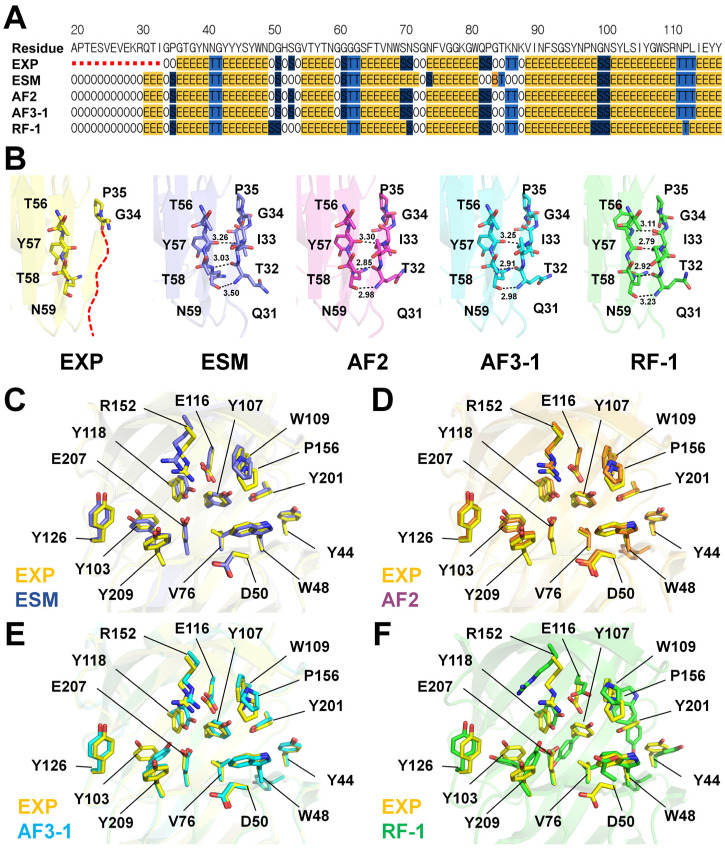
Structural comparison of the experimental and AI-predicted HviGH11 models. (**A**) Secondary structure analysis of the experimental and AI-predicted HviGH11 models. (**B**) Close-up view of the N-terminal region of EXP-, AF2-, ESM-, AF3-, and RF-HviGH11. The experimentally disordered N-terminal region of EXP-HviGH11 is shown as a red dotted line. Superimposition of EXP-HviGH11 with (**C**) ESM-, (**D**) AF2-, (**E**) AF3-, and (**F**) RF-HviGH11 models.

To assess the structural differences between the experimental and AI-predicted structures, the experimental structure of HviGH11 (EXP-HviGH11) was compared with its AI-predicted models. Superimposition of EXP-HviGH11 with the predicted models revealed RMSDs of 0.87 Å for ESM, 0.30 Å for AF2, 0.30–0.42 Å for AF3, and 0.77–0.88 Å for RF. (Supplementary Figure S4). The N-terminal region from Ala20 to Ile33 of EXP-HviGH11 was disordered in the electron density map (Supplementary Figure S5). Secondary structure analysis of EXP-HviGH11 revealed that the first β-sheet (Gly36–Asn40) began from Gly36 (Figure 3A,B). Conversely, in all AI-predicted HviGH11 models, the first β-sheet (Gln31–Ile33) began from Gln31 (Figure 3A and Supplementary Figure S6), a region that was disordered in the experimental structure. In these models, the main chains of Gln31, Thr32, and Ile33 formed hydrogen bonds with the main chains of Tyr57, Thr58, and Asn59 on the adjacent β-sheet (Figure 3B).

Superimposition of EXP-HviGH11 with ESM-HviGH11 revealed identical side-chain conformations for most substrate-binding and catalytic residues, excluding Val76 and Ser86 (Figure 3C). Superimposition of EXP-HviGH11 and AF2-HviGH11 demonstrated identical conformations of residues involved in the substrate-binding cleft (Figure 3D). Superimposition of EXP-HviGH11 and AF3-HviGH11 models highlighted differences in the side-chain conformations at Val76, Tyr103, and Glu207 across the five predicted models (Figure 3E and Supplementary Figure S7). Superimposition of EXP-HviGH11 and RF-HviGH11 models revealed significant conformational differences in the catalytic residues Glu116 and Glu207. (Figure 3F and Supplementary Figure S7). In addition, 12 residues located in the substrate-binding cleft, namely Tyr44, Trp48, Asp50, Val76, Tyr107, Trp109, Tyr118, Tyr126, Arg152, Ile158, Tyr201, and Tyr209, exhibited substantially different conformations across the five RF-HviGH11 models (Figure 3F and Supplementary Figure S7). Furthermore, none of the five RF-HviGH11 structures exhibited identical conformation of the substrate-binding residues when compared with EXP-HviGH11.

**Figure 4:**
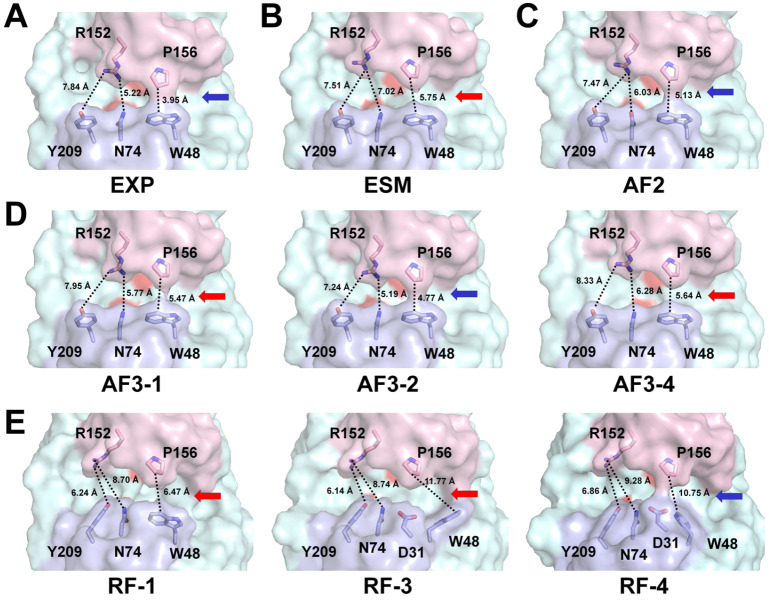
Surface representations of experimental and AI-predicted HviGH11 models. Surface structure of the (**A**) closed conformation of EXP-HviGH11, (**B**) open conformation of ESM-HviGH11, (**C**) closed conformation of AF2-HviGH11, (**D**) open and closed conformations of AF3-HviGH11, and (**E**) open and closed conformations of RF-HviGH11. The open and closed conformations between the finger (light blue) and thumb (light pink) domains are indicated by red and blue arrows, respectively.

In GH11 xylanases, the positions of the thumb and finger domains affect the width of the substrate-binding cleft, resulting in either an open or a closed conformation [38,39]. Specifically, open loop conformations increase the size of the substrate-binding cleft and facilitate substrate access, whereas closed or compact conformations restrict the cleft and position catalytic residues to optimize catalysis [40,41,42]. The width of the substrate-binding cleft of EXP-HviGH11 differed notably from those of the AI-predicted models (Table S1). In EXP-HviGH11, the closest distance between the CZ2 atom of Trp48 located on the finger domain and the CG atom of Pro156 on the thumb domain was 3.95 Å, revealing a closed conformation in the surface structure (Figure 4A). The distance between the ND2 atom of Asn74 and the NH2 atom of Arg152, located at subsite +1, was 5.22 Å (Figure 4A). The domain angle between the three Cα atoms of Trp48 (finger domain), Glu188 (palm domain), and Pro156 (finger domain) of EXP-HviGH11 was 44.4°. The calculated volume of the substrate-binding cavity was 909 Å^3^. In ESM-HviGH11, the thumb domain was shifted away from the substrate-binding cleft compared to that in EXP-HviGH11. The closest distance between Trp48 and Pro156 was 5.47 Å, which exceeded that observed in EXP-HviGH11, displaying a slightly open conformation between the thumb and finger domains. The distance between Asn74 and Arg152 was 8.06 Å, reflecting a significant deviation in the side-chain orientation of Arg152 relative to EXP-HviGH11. The domain angle between the three Cα atoms of Trp48, Glu188, and Pro156 of ESM-HviGH11 was 48.7°. The calculated cavity volume was 1046 Å^3^, approximately 15% larger than that of EXP-HviGH11 (Figure 4B). In AF2-HviGH11, the closest distance between Trp48 and Pro156 was 5.13 Å, exhibiting a relatively closed conformation (Figure 4C). The distance between Asn74 and Arg152 was 6.03 Å. Although the overall surface conformation of AF2-HviGH11 was similar to that of EXP-HviGH11, the substrate-binding cleft appeared narrower. The domain angle between the three Cα atoms of Trp48, Glu188, and Pro156 of AF2-HviGH11 was 46.3°. The calculated cavity volume was 812 Å^3^, approximately 10% smaller than that of EXP-HviGH11. In the five AF3-HviGH11 models, the distances between the thumb and finger domains varied because of subtle differences in the side-chain orientations of substrate-binding residues and positional differences in the thumb domains. The distances between Trp48 and Pro156 in models 1–5 were 5.47, 4.77, 4.93, 5.64, and 5.16 Å, respectively, all exceeding the corresponding distance observed in EXP-HviGH11 (Figure 4D and Supplementary Figure S8). The distances between Asn74 and Arg152 in models 1–5 were 5.77, 5.19, 5.54, 6.28, and 5.67 Å, respectively, indicating variable degrees of cleft opening at subsite +1. The domain angles between the three Cα atoms of Trp48, Glu188, and Pro156 of AF3-HviGH11 models 1, 2, 3, 4, and 5 were 45.6°, 45.5°, 45.7°, 46.9°, and 45.3°, respectively. Surface structure analysis revealed that closed cleft conformations of three of the AF3 models were similar to that observed in EXP-HviGH11. Conversely, one model displayed a more open and one model exhibited a more closed conformation of the substrate-binding cleft. The calculated substrate-binding cavity volumes for AF3 models 1–5 were 886, 896, 796, 961, and 818 Å^3^, respectively. In the five RF-HviGH11 models, the distances between the thumb and finger domains varied significantly because of conformational differences in the substrate-binding residues. In model 1, the distance between Trp48 and Pro156 was 6.47 Å (Figure 4E). However, in models 2–5, the side chain of Trp48 rotated toward the palm domain, resulting in a substantial structural difference from that observed in EXP-HviGH11 (Figure 4E and Supplementary Figure S8). The distances between Trp48 and Pro156 for models 2–5 ranged from 9.17 Å to 11.77 Å. Additionally, the side chain of Arg152 was directed toward subsite +2, in contrast to its orientation toward the finger domain in EXP-HviGH11. The distances between Asn74 and Arg152 in RF-HviGH11 models 1–5 were 8.70, 9.63, 8.74, 9.28, and 8.93 Å, respectively (Figure 4E and Supplementary Figure S8). Among the five models, only model 4 displayed a closed conformation of the substrate-binding cleft similar to EXP-HviGH11, whereas the other models exhibited open conformations. The domain angles between the three Cα atoms of Trp48, Glu188, and Pro156 of RF-HviGH11 models 1, 2, 3, 4, and 5 were 52.1°, 50.9°, 49.6°, 52.3°, and 50.9°, respectively. The calculated substrate-binding cavity volumes for models 1–5 were 1346, 890, 838, 1133, and 500 Å^3^, respectively.

### 2.4. Docking of Xylohexaose to the Experimental and AI-Predicted Models of HviGH11

Ligand docking to the target protein offers insights into the molecular mechanisms of the proteins and drug design [43,44,45]. The ligand docking results are highly dependent on the model protein structure [46]. Because of positional differences in the thumb and finger domains, as well as catalytic and substrate-binding residues, between the experimental and AI-predicted models, the subsequent docking results also varied. To understand the differences in the docking outcome between the experimental and AI-predicted structures, xylohexaose was used as the ligand and docked into the HviGH11 structures using an automated docking program to avoid bias (Table S2).

**Figure 5:**
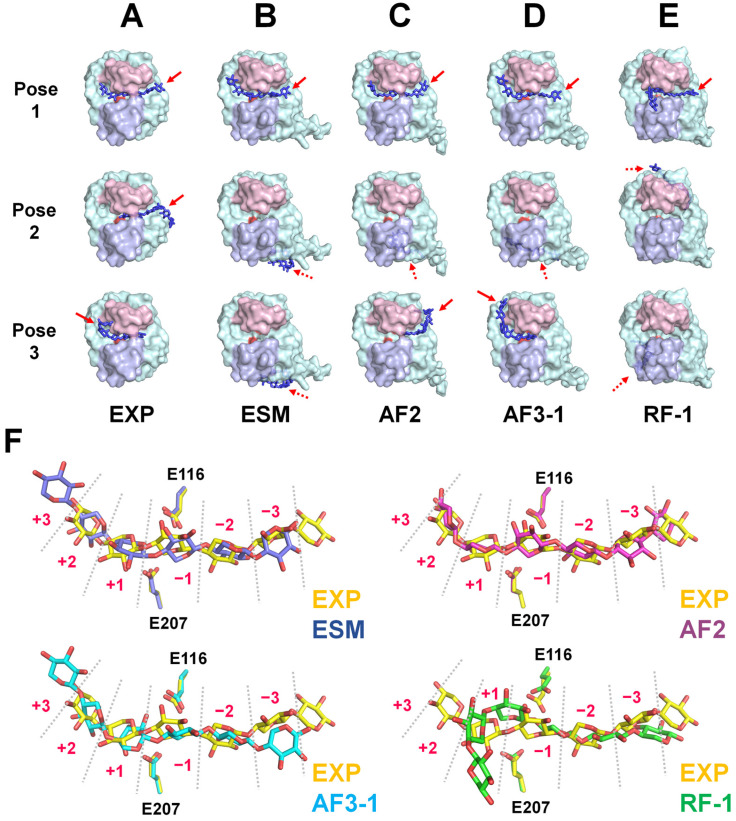
Xylohexaose docking to the experimental and AI-predicted HviGH11 structures. Surface representation of xylohexaose (blue sticks) docked to (**A**) EXP-HviGH11, (**B**) ESM-HviGH11, (**C**) AF2-HviGH11, (**D**) AF3-HviGH11 (AF3-1), and (**E**) RF-HviGH11 (RF-1). The finger and thumb domains are indicated by light pink and light blue surfaces, respectively. The catalytic residues are shown in red. The red arrow with a solid line indicates the substrate position at the side of the substrate-binding cleft, whereas the red arrow with a dotted line indicates the substrate position at the opposite side of the substrate-binding cleft. (**F**) Close-up views of the superimposition of xylohexaose bound to EXP-HviGH11 (yellow) with ESM-HviGH11 (blue), AF2-HviGH11 (purple), AF3-HviGH11 (AF3-1, cyan), and RF-HviGH11 (RF-1, green).

In EXP-HviGH11, pose 1 of xylohexaose, the highest-scoring docking pose, exhibited occupancy of five subsites (+2 to −3) within the HviGH11 substrate-binding cleft, with a predicted binding affinity of −12.5 kcal/mol (Figure 5A). Three xylose units in pose 2 and four xylose units in pose 3 were bound at subsites −1 to −3 and +2 to −2, respectively (Figure 5A). In ESM-HviGH11, pose 1 of xylohexaose exhibited six xylose units from xylohexaose occupying all subsites (+3 to −3), with a predicted binding affinity of −12.1 kcal/mol (Figure 5B). Poses 2 and 3 were docked outside the substrate-binding cleft of ESM-HviGH11 (Figure 5B). In AF2-HviGH11, pose 1 of xylohexaose exhibited occupancy of five subsites (+2 to −3) within the HviGH11 substrate-binding cleft, with a predicted binding affinity of −12.5 kcal/mol (Figure 5C). Meanwhile, pose 2 was docked outside the substrate-binding cleft. Two xylose units in pose 3 were bound at subsites −2 and −3 (Figure 5C). In the highest-scoring AF3-HviGH11 model 1, pose 1 of xylohexaose displayed full occupancy of all six subsites (+3 to −3) within the HviGH11 substrate-binding cleft, with a predicted binding affinity of −12.8 kcal/mol. Pose 2 docked outside of the substrate-binding site (Figure 5D). Three xylose units in pose 3 were docked to subsites +2 to −1 of AF3-HviGH11 (Figure 5D). In the highest-scoring RF-HviGH11 model 1, four xylose units in pose 1 of xylohexaose occupied four subsites (+1 to −3), with a predicted binding affinity of −11.0 kcal/mol (Figure 5E). Poses 2 and 3 docked outside of the substrate-binding site (Figure 5E).

Next, the AI-predicted models of HviGH11 were superimposed onto the experimental structure, and the positions and conformations of the docked xylohexaose molecules (pose 1) were compared. The position of the xylose units in xylohexaose docked in EXP-HviGH11 and AF2-HviGH11 was similar, whereas the positions of xylohexaose docked in ESM-HviGH11 and AF3-HviGH11 were shifted by approximately one xylose unit toward the +3 subsite compared with EXP-HviGH11 (Figure 5F). Although the docked xylohexaose molecules in all of the HviGH11 models were located on the substrate-binding cleft, the detailed positions and conformations of the individual xylohexaose molecules varied across the models. The closest distances from the OE atoms of Glu207/Glu97 to the glycosidic oxygen between the xylose units at subsites +1 and −1 were 4.87/3.80 Å for EXP-HviGH11, 4.76/3.36 Å for ESM-HviGH11, 5.39/3.16 Å for AF2-HviGH11, and 5.19/3.66 Å for AF3-HviGH11. These results indicate that subtle differences in the positions of substrate-binding and catalytic residues between EXP-HviGH11 and the AI-predicted models generated by ESM, AF2, and AF3 can influence the binding orientation of the ligand within the substrate-binding cleft. In contrast, most of the xylose units in the xylohexaose docked to RF-HviGH11 were not located within the substrate-binding cleft, and both the position and conformation of the ligand differed significantly from those in the other models (Figure 5F). These distinct docking results are likely due to inaccurate conformations of the substrate-binding and catalytic residues in the RF-HviGH11 model.

## 3. Discussion

Recent advances in AI-based protein structure prediction tools, such as AF2, AF3, ESM, and RF, have significantly enhanced the accessibility of structural information for biochemical and molecular biology studies. These models are widely used in protein engineering and drug design, especially when experimental structures are unavailable. However, when AI-predicted models are inaccurate or fail to capture the native conformation, they can lead to the misinterpretation of the molecular functions and potentially misguide downstream applications, such as drug design. Therefore, critical evaluation of AI-predicted models is essential for both fundamental research and industrial applications.

In this study, the previously unreported crystal structure of HviGH11 was compared with AI-predicted models generated using ESM, AF2, AF3, and RF. All the predicted structures retained the overall β-jelly roll fold characteristic of GH11 xylanases; however, significant structural differences were observed in the N-terminal region. The N-terminal region of GH11 xylanases is known to influence their enzymatic activities and thermostabilities, and it is frequently targeted for protein engineering [47,48]. In all AI-predicted models, three residues in the N-terminal region (Gln31–Ile33) formed hydrogen bonds with the neighboring β-strand, resulting in a stable β-sheet. In contrast, the N-terminal region in the EXP-HviGH11 structure was disordered in the electron density map, suggesting high flexibility or an inability to form a defined secondary structure under crystallographic conditions. This discrepancy could result in divergent interpretations regarding the structural stability and flexibility of the N-terminal of HviGH11, particularly if used to guide N-terminal engineering strategies. This result indicated that AI-predicted models might exhibit inaccurate protein folding for the flexible regions of the protein. Meanwhile, crystallization conditions can influence the conformational behavior of flexible protein regions. Accordingly, future structural determination under alternative crystallization conditions will be useful to assess whether the N-terminal flexibility of HviGH11 is an intrinsic property of the protein or exhibits condition-dependent modulation.

AI-predicted models displayed varying similarity to the experimental structure, particularly in the substrate-binding region and active sites. The ESM and AF2 models exhibited the greatest similarity with the experimental structure in terms of conformations of the substrate-binding and active sites. Among the five AF3-predicted models, only three models were highly similar to the experimental structure, whereas the remaining two models displayed significantly different side-chain orientations in the substrate-binding and active site residues, potentially resulting in different interpretations in structural analysis. AF3 ranks its models based on pTM scores. However, the results suggested that these scores do not always correlate with the degree of structural similarity to the experimental model. Thus, the selection of models from a set of AI predictions can influence downstream structural analyses. Notably, the five models generated by RF exhibited considerable inconsistencies in the orientation of key substrate-binding residues. These conformations differed among the five RF-predicted models and from the corresponding conformation observed in the experimental structure. If the substrate-binding and active sites of HviGH11 had been interpreted according to the RF-predicted models, it would have resulted in an inaccurate structural analysis. RF has provided reliable structural information in the prediction of various proteins [22,49]. However, the inaccurate structural features observed in the current study might be attributed to the unsuitability of RF for modeling HviGH11 owing to a general limitation of the algorithm.

Based on the conformation of thumb and finger domains, EXP-HviGH11 exhibited a narrower substrate-binding cleft compared to the AI-predicted models. Considering that GH11 xylanases can adopt either open or closed conformations between the thumb and finger domains depending on crystal packing, environmental conditions, data collection method, or substrate binding [38,39,50,51,52], the broader cleft predicted by the AI models does not necessarily indicate an inaccurate structure. Nevertheless, the AI-predicted models failed to capture the narrower, closed conformation observed in EXP-HviGH11, highlighting a limitation of current structure prediction methods in revealing the full range of conformational variability of HviGH11.

Ligand docking studies are important for understanding molecular mechanisms or drug design, and docking results are directly influenced by the structure of the model used for the target protein [53,54]. In the current study, structural differences in the side-chain orientation of the substrate-binding site and domain positioning between the experimental and AI-predicted structures affected the substrate-binding cavity, as well as the docking simulations, because of subtle structural differences. In previously reported crystal structures of xylanase GH11 in complex with oligoxylose, the xylose units are located on the substrate-binding cleft. Similarly, in the high-scoring docking poses of xylohexaose onto EXP-, ESM-, AF2-, and AF3-HviGH11 models, the xylose units consistently occupy more than five subsites within the substrate-binding cleft. In contrast, the xylohexaose docked onto the RF-HviGH11 model binds to only four subsites and exhibits a significantly different configuration from the other models because of incorrect side-chain conformations of the residues involved in the substrate-binding cleft. This xylohexaose binding mode does not align with those previously reported for xylanase GH11, which could lead to inaccurate functional interpretation for substrate recognition and enzyme function. These findings highlight that the choice of an AI-predicted model can significantly affect downstream research, underscoring the importance of selecting a reliable and appropriate prediction model.

Furthermore, the detailed binding positions and configurations of xylohexaose differ among the EXP-, ESM-, AF2-, and AF3-HviGH11 models. These differences likely arise from subtle variations in the conformations or positions of substrate-binding and catalytic residues, leading to distinct substrate-binding modes. Since the experimental structure of xylohexaose-bound HviGH11 has not yet been determined, it is currently not possible to assess which model provides the most accurate docking pose. Accordingly, this study highlights that variations in protein model structures can lead to differences in ligand binding, which may, in turn, result in divergent interpretations of research outcomes. To determine which model most accurately represents the ligand-bound conformation, it will be necessary to experimentally determine the crystal structure of HviGH11 in complex with xylohexaose in future studies. In addition, further studies will be required to determine whether energy minimization or molecular dynamics simulations can reduce structural discrepancies between experimental and AI-predicted models and improve their conformational consistency.

Taken together, these conclusions underscore the importance of selecting an appropriate model structure for ligand docking studies and highlight the potential impact of structural inaccuracies in AI-predicted models. If the model structure or the resulting ligand docking pose is incorrect, it could lead to flawed research strategies and substantial negative consequences for subsequent studies. Therefore, when utilizing AI-predicted models, it is essential to first assess whether the provided structure is reliable. This assessment requires a prior understanding of protein folding and structural features of homologous proteins, as well as structural expertise to determine whether the predicted model is reasonable.

## 4. Materials and Methods

### 4.1. Structure Determination

Protein preparation and data collection were performed as previously described [25,37]. Briefly, the coding sequence of HviGH11 (UniProt ID: G9MJY8), excluding the signal peptide, was cloned into the pHBA vector (Bioneer, Daejeon, Republic of Korea). The recombinant protein was expressed in *Escherichia coli* BL21 (DE3) and purified via Ni-NTA affinity chromatography. The purified protein was concentrated to approximately 30 mg/mL and mixed with an equal volume of 10% (*w*/*v*) PEG4000. Crystallization was conducted by incubating the mixtures at 4 °C overnight. X-ray diffraction data were collected at 100 K at beamline 11C of the Pohang Accelerator Laboratory (Pohang, Republic of Korea). The diffraction data were processed using HKL2000 [26]. The structure was solved via molecular replacement using MOLREP [55] implemented in the CCP4 suite [56]. The crystal structure of xylanse II from Trichoderma longibrachiatum (PDB code: 8YJJ) [51] was used as a search model. The model was built using COOT [57] and refined using phenix.refine in the PHENIX package [58]. The quality of the final structure was assessed using MolProbity [59].

### 4.2. Generation of the AI-Predicted Structure

Prediction of HviGH11 by ESM [23] was performed using the ESM Metagenomic Atlas (https://esmatlas.com/resources?action=fold, accessed on 1 July 2025). The structure predicted by AF2 [20] was downloaded from the AlphaFold Protein Structure Database (https://alphafold.ebi.ac.uk/entry/G9MJY8, accessed on 1 July 2025). HviGH11 structure prediction by AF3 [21] was performed on the AF3 server (https://golgi.sandbox.google.com/, accessed on 1 July 2025). Structure prediction by RF [22] was performed using the Robetta protein structure prediction service (https://robetta.bakerlab.org/, accessed on 1 July 2025). In the structure prediction using ESM, AF3, and RF, the amino acid sequence of HviGH11 without the signal peptide was used as input. All models were generated using default parameters.

### 4.3. Bioinformatics

Structure alignments were performed using TM-align [60]. The secondary structures of both the AI-predicted and experimental structures were compared using 2StrucCompare [61] based on the Dictionary of Secondary Structure of Proteins [62]. The substrate binding pocket and ligand docking were analyzed using CB-Dock2 [63], which automatically detects potential binding cavities on the protein surface and performs docking using the AutoDock Vina engine (v1.2.7). The coordinates of the docking substrate model for xylohexaose were obtained from the crystal structure of GH11 xylanase from *Trichoderma reesei* (PDB code: 4HK8). During molecular docking for AF3 and RF, the highest-scoring model was selected from multiple predicted models based on the confidence metrics provided by each tool (pTM score for AF3 and RMSD-based estimates for RF). Structural figures were prepared using PyMOL (version 2.4.1, DeLano Scientific LLC, San Carlos, CA, USA).

## 5. Conclusions

In this study, the reliability of AI-predicted models was evaluated by comparing them with the previously unreported crystal structure of HviGH11. All AI-predicted structures retained the canonical β-jelly roll fold of GH11 xylanases; however, significant differences were observed in the N-terminal region and the substrate-binding sites. Although some AI-predicted models displayed high structural similarity to the experimental model, others, particularly those generated by RF, failed to replicate critical local conformations. The intrinsic flexibility of the thumb and the finger domains of GH11 xylanases potentially contributes to structural variability, influencing the shape of the substrate-binding cleft and the accuracy of docking predictions. These results demonstrated that relying solely on AI-predicted models can lead to inaccurate conclusions regarding protein function or ligand interactions. Therefore, integrating experimental data with AI-based predictions and cross-validating models using multiple tools is crucial for robust structural and functional analysis. These results offer valuable insights into the appropriate use of AI-predicted structures in biochemical research and provide a practical approach for their application in drug discovery and protein engineering.

## Figures and Tables

**Table 1:** Structure refinement statistics of HviGH11.

Data Collection	HviGH11
Space group	P2_1_2_1_2_1_
Unit cell (Å) a, b, c	43.33, 51.27, 94.44
Resolution (Å)	50.00–1.95 (1.98–1.95)
Unique reflections	15,684 (778)
Completeness (%)	97.6 (97.7)
Redundancy	5.0 (4.7)
Mean *I*/σ (*I*)	9.00 (1.90)
R_merge_	0.130 (0.595)
CC1/2	0.993 (0.811)
CC*	0.998 (0.946)
Structure refinement	
Resolution (Å)	45.06–1.95 (1.98–1.95)
R_work_^a^	0.1700 (0.1915)
R_free_^b^	0.2018 (0.2311)
R.m.s. deviations	
Bonds (Å)	0.006
Angles (°)	0.846
*B* factors (Å^2^)	
Protein	19.65
Water	31.39
Ramachandran plot (%)	
Favored	98.38
Allowed	1.62
Disallowed	0.00
PDB code	9VXQ

Values for the outer shell are given in parentheses. ^a^ R_work_ = Σ||F_obs_| − |F_calc_||/Σ|F_obs_|, where Fobs and F_calc_ are the observed and calculated structure factor amplitudes, respectively. ^b^ R_free_ was calculated as R_work_ using a randomly selected subset (10%) of unique reflections not used for structural refinement. The data collection statistics were obtained from Ref. [37], which reported the crystallization experiments and data collection.

## Data Availability

The structure factors and coordinates of HviGH11 were deposited in the Protein Data Bank under accession code 9VXQ. The model structures generated by AI tools and the xylohexaose docking results have been deposited in Zenodo (https://doi.org/10.5281/zenodo.16196298).

## Supplementary Materials

The following supporting information can be downloaded at: https://www.mdpi.com/article/10.3390/ijms27031370/s1.

## Abbreviations

The following abbreviations are used in this manuscript:

AI	Artificial intelligence
ESM	ESMFold
AF2	AlphaFold2
AF3	AlphaFold3
RF	RoseTTAFold
