# Supplementary material for: Evaluation of AI-Predicted GH11 Xylanase Models Against a Previously Unreported Experimental Structure: Implications for Conformational Accuracy and Ligand Binding
Source: Int J Mol Sci. 2026 Jan 29;27(3):1370. doi: 10.3390/ijms27031370 (PMC12897616; doi:10.3390/ijms27031370)
Supplement: Supplementary file 1 [file ijms-27-01370-s001.zip › ijms-4103930-supplementary.pdf]

Supplementary Data

Evaluation of AI-Predicted GH11 Xylanase Models Against a Previously Unreported Experimental Structure: Implications for Conformational Accuracy and Ligand Binding

Ki Hyun Nam

College of General Education, Kookmin University, Seoul 02707, Republic of Korea

*Corresponding: structure@kookmin.ac.kr

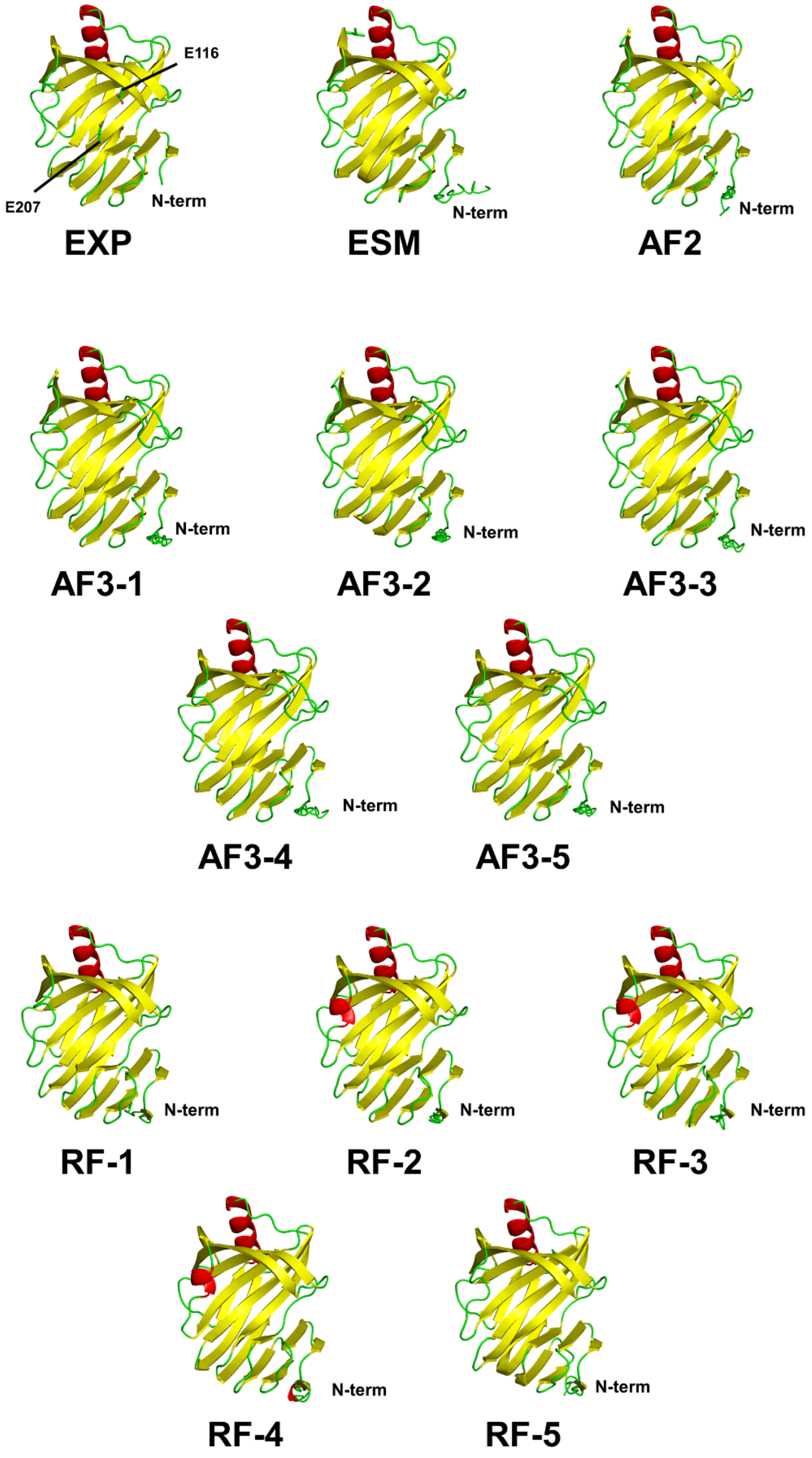

Supplementary Figure S1. AI-predicted structures of HviGH11 generated by ESMFold, AlphaFold2, AlphaFold3, and RoseTTAFold.

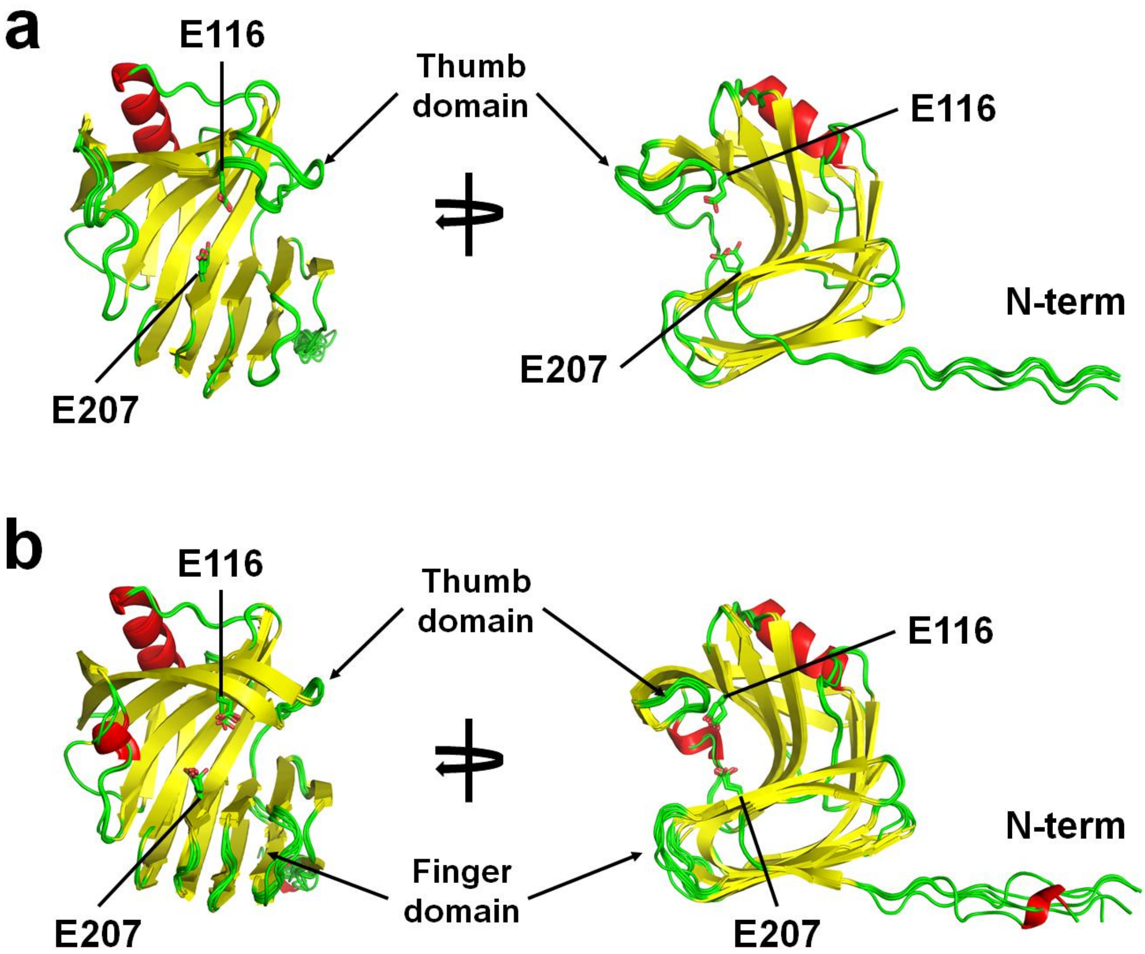

Supplementary Figure S2. Superimposition of five HviGH11 models predicted by (a) AlphaFold3 and (b) RoseTTAFold.

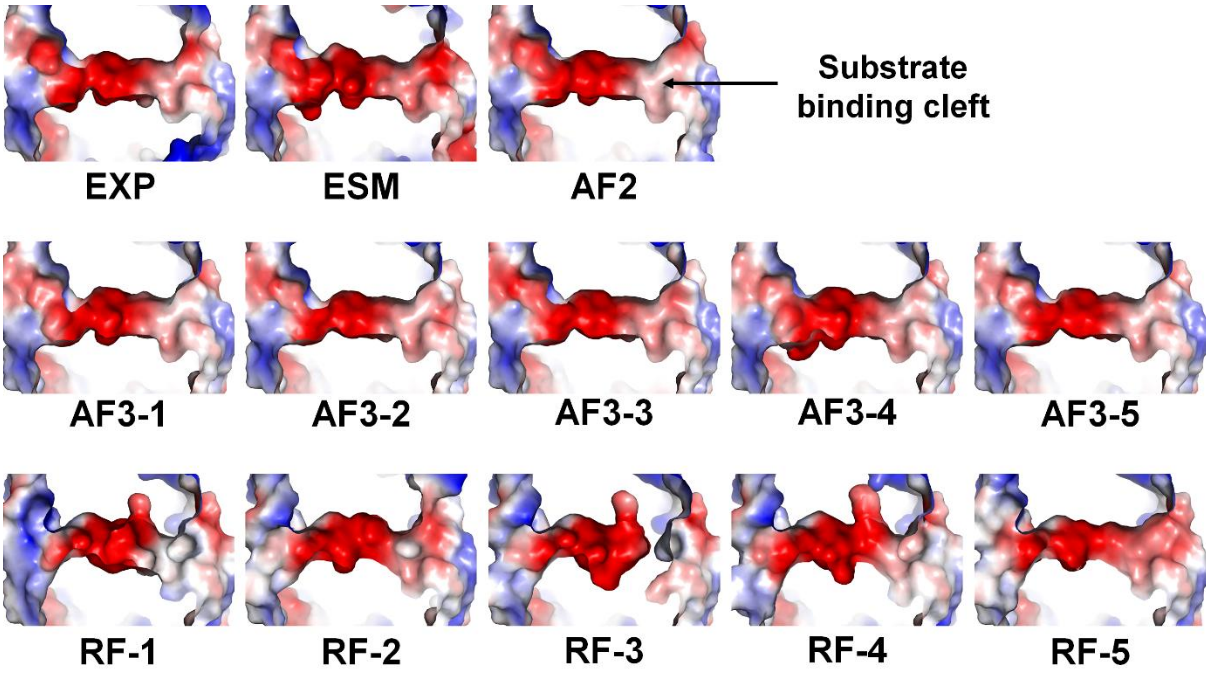

Supplementary Figure S3. Electrostatic surfaces of the substrate-binding cleft in the experimental HviGH11 structure (EXP) and AI-predicted models generated by ESMFold (ESM), AlphaFold2 (AF2), AlphaFold3 (AF3), and RoseTTAFold (RF).

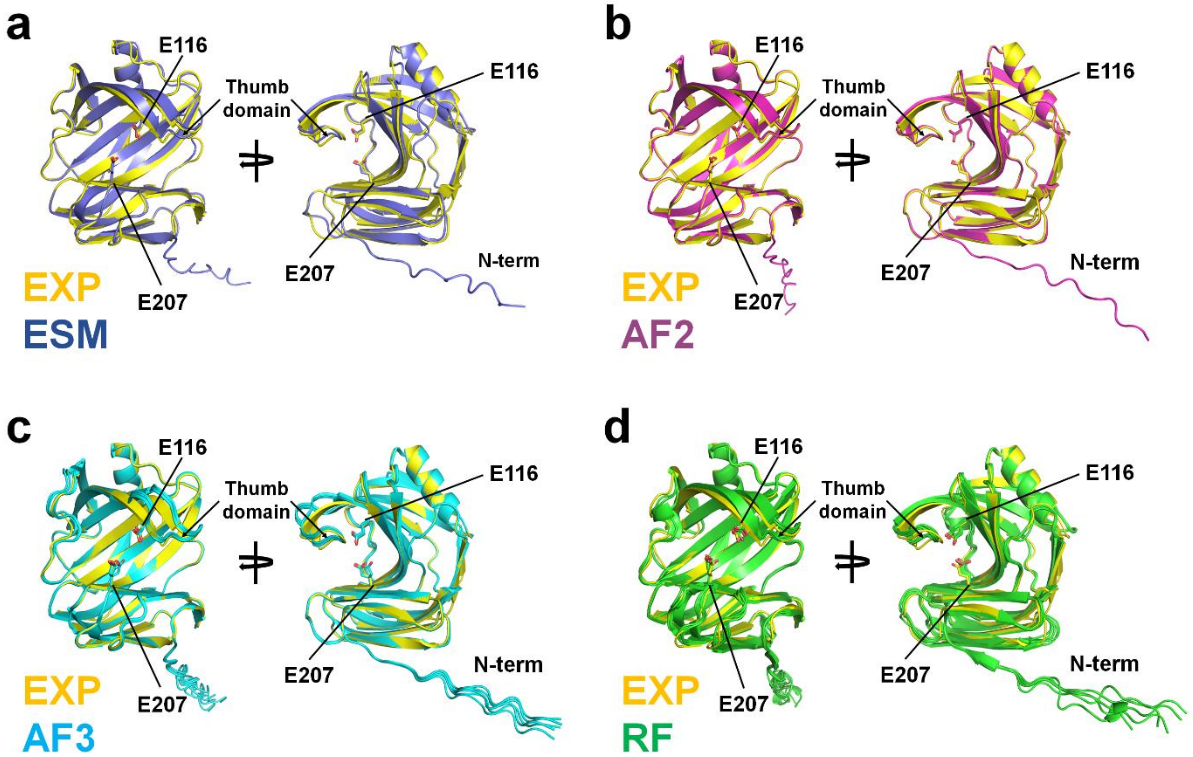

Supplementary Figure S4. Superimposition of the experimental HviGH11 structure (EXP, yellow) with AI-predicted models generated by (a) ESMFold (ESM, blue), (b) AlphaFold2 (AF2, purple), (c) AlphaFold3 (AF3, cyan), and (d) RoseTTAFold (RF, green).

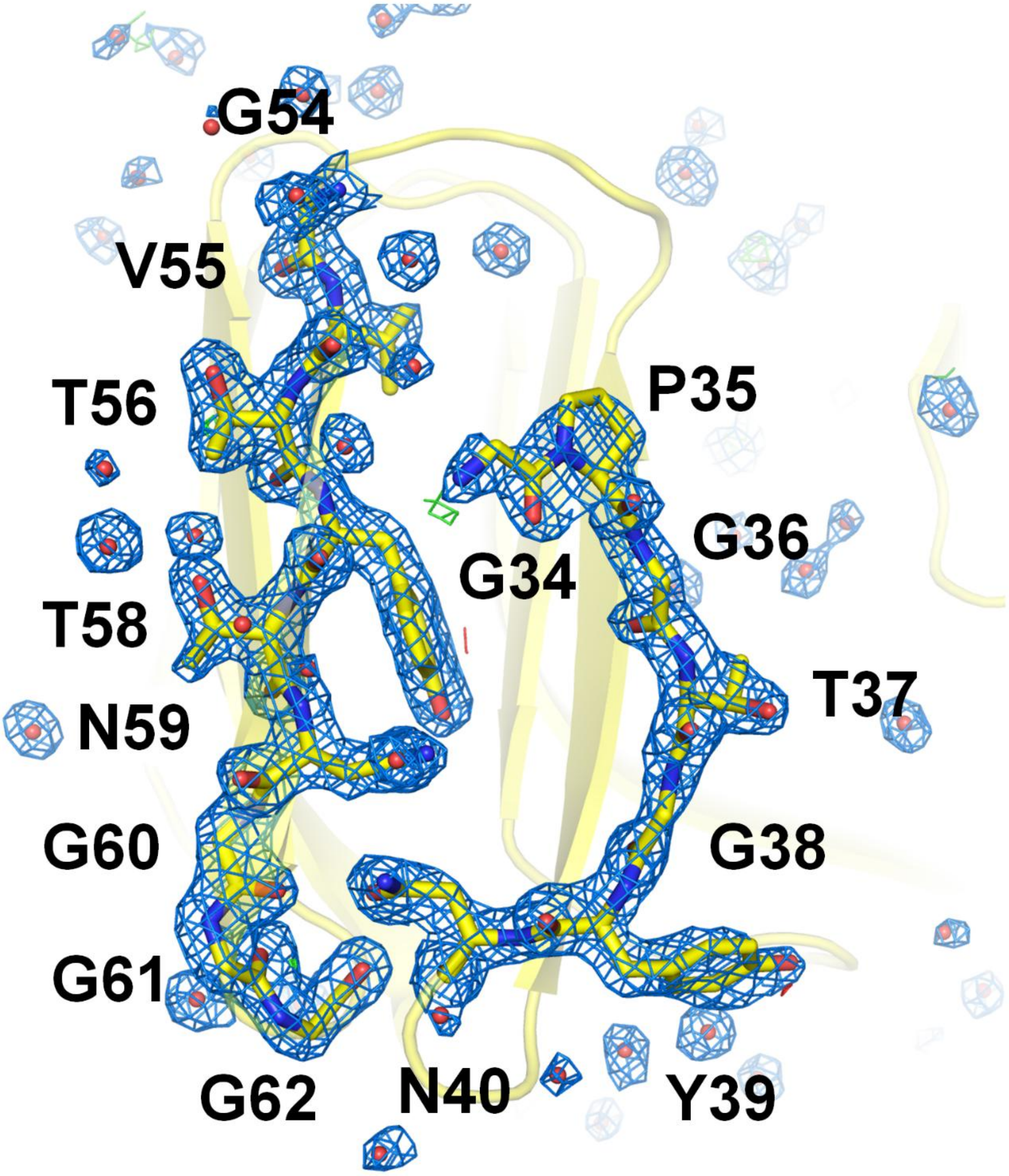

Supplementary Figure S5. The 2Fo–Fc (blue mesh, 1σ) and Fo–Fc (green mesh, +3σ; red mesh, –3σ) electron density maps for the N-terminal region of the HviGH11 crystal structure.

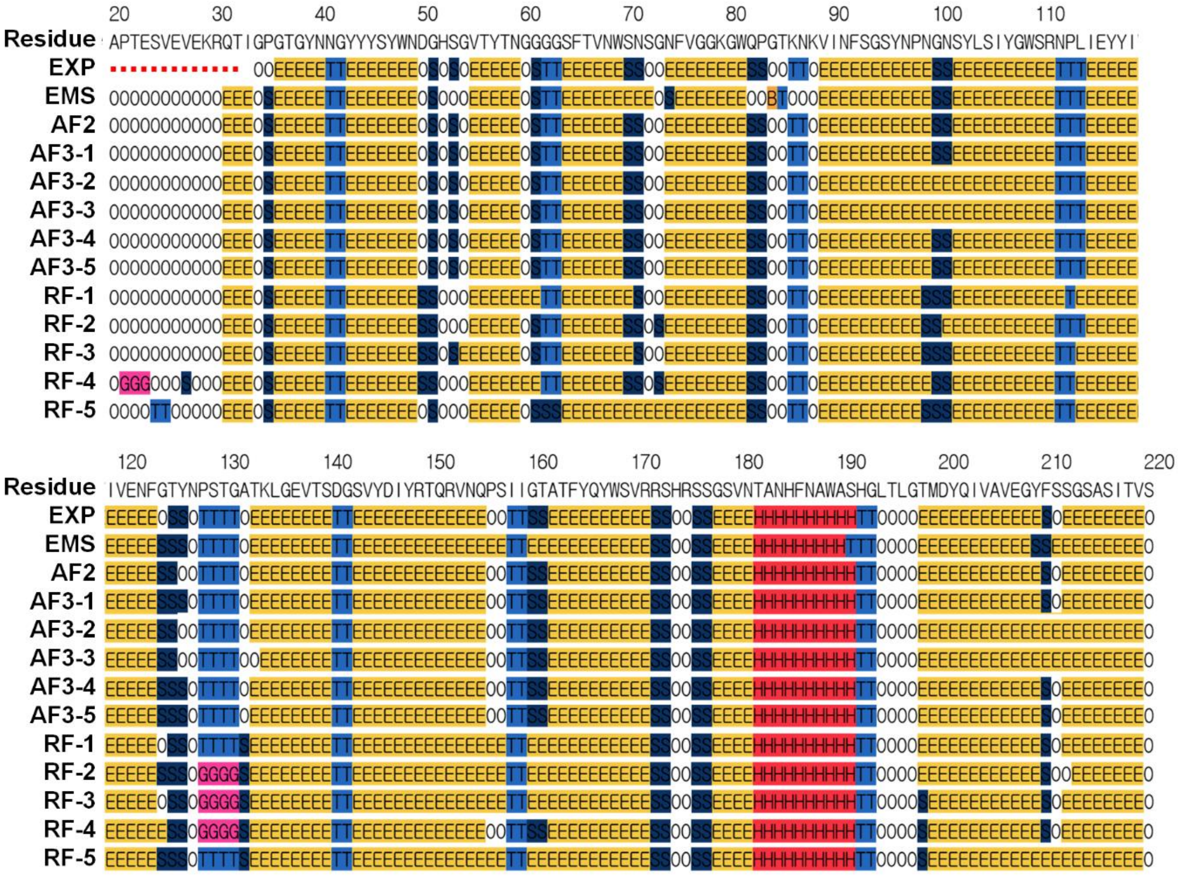

Supplementary Figure S6. Secondary structure analysis of the experimental (EXP) and AI-predicted models of HviGH11 generated by ESMFold (ESM), AlphaFold2 (AF2), AlphaFold3 (AF3), and RoseTTAFold (RF). Each residue is annotated using the standard DSSP notation: H (α-helix), G (3_{10}-helix), E (extended β-strand), B (isolated β-bridge), T (hydrogen-bonded turn), S (bend), and 0 (loop or coil).

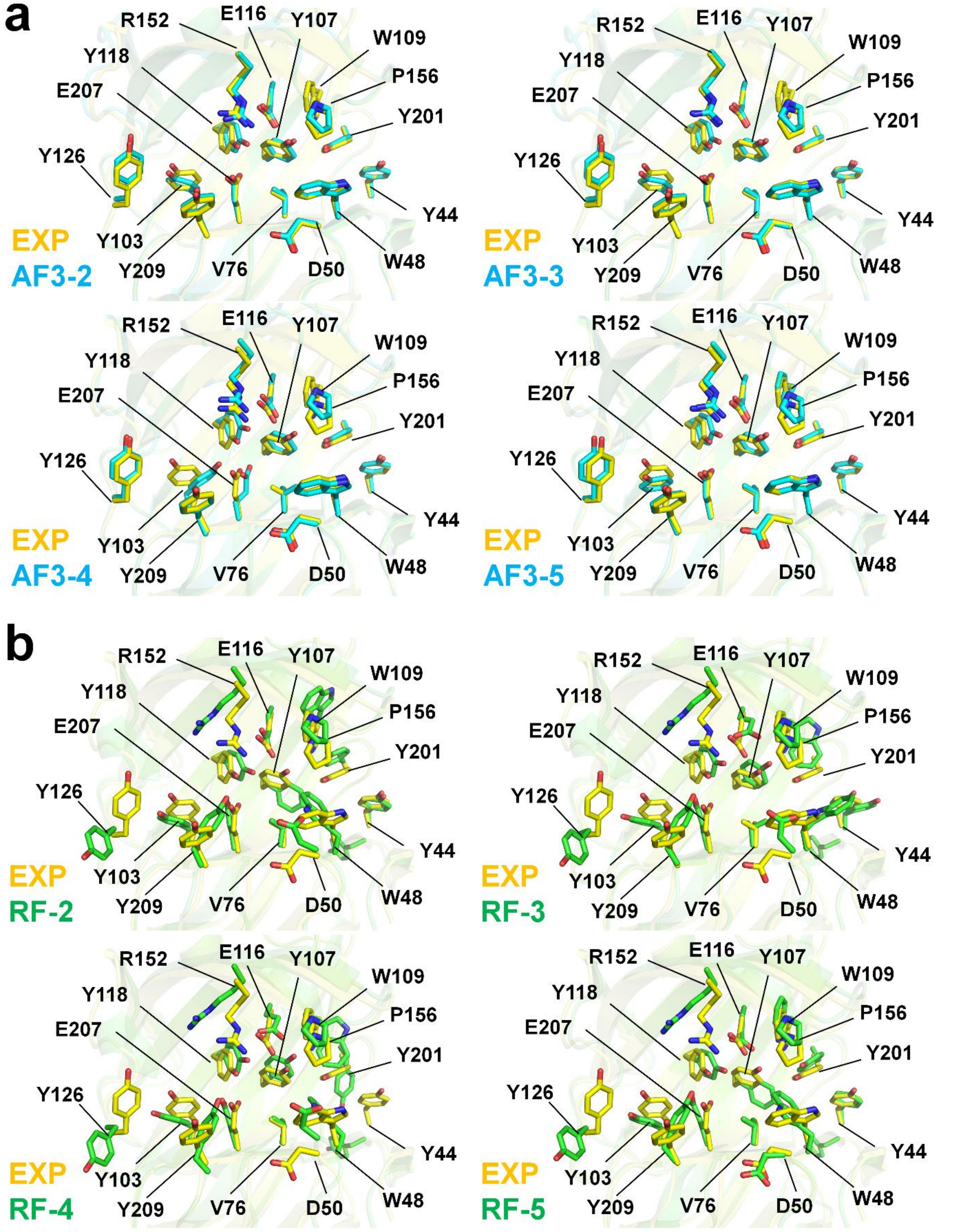

Supplementary Figure S7. Superimposition of the experimental HviGH11 (EXP, yellow) with models predicted by (a) AlphaFold3 (AF3, cyan) and (b) RoseTTAFold (RF, green).

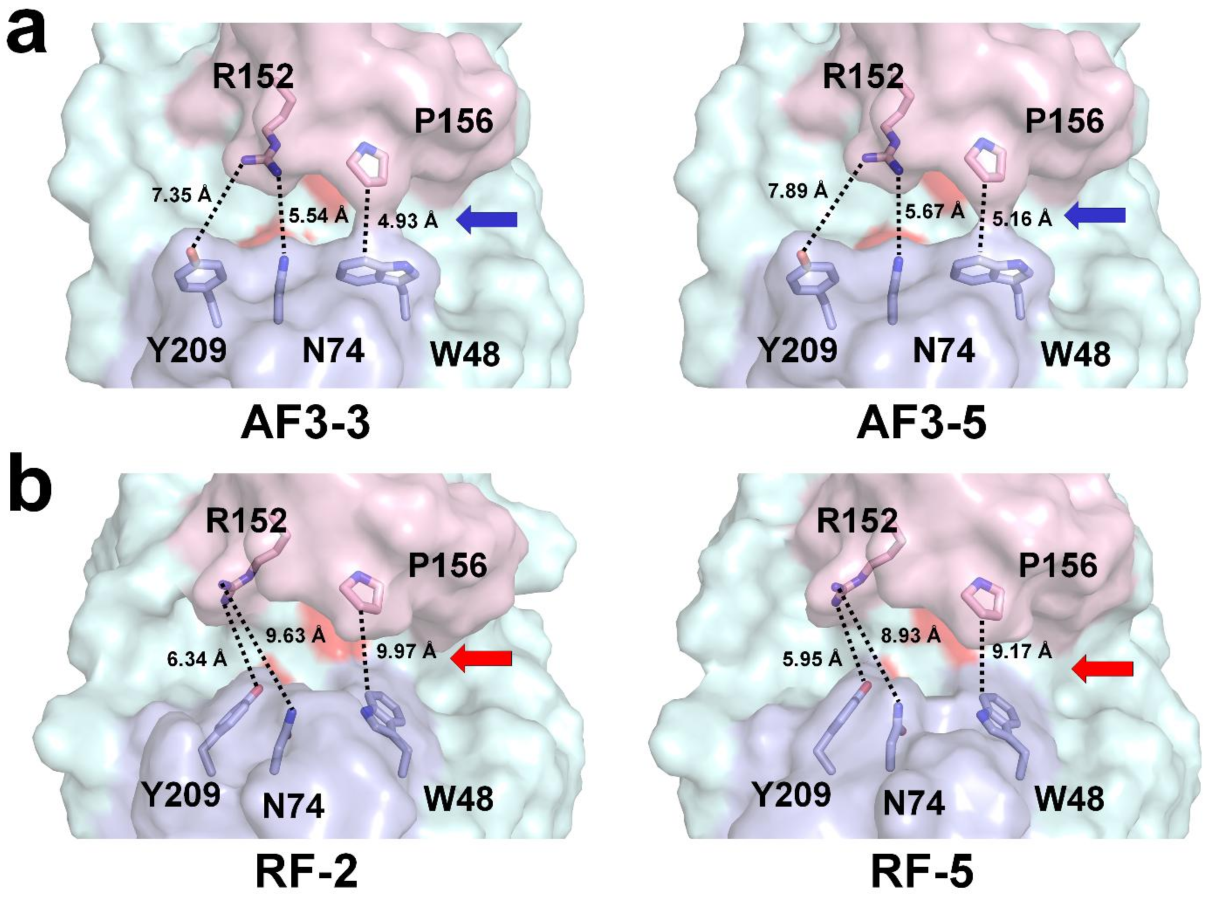

Supplementary Figure S8. Surface structures of HviGH11 predicted by (a) AlphaFold3 and (b) RoseTTAFold. The open and closed conformations between the finger (light blue) and thumb (light pink) domains are indicated by red and blue arrows, respectively.

Table S1. Summary of structural comparison between experimental and AI-predicted HviGH11.

Model	Substrate-binding cleft conformation	Thumb–finger domain distance (Å)			Domain angle (°) (W48- E188-P156)	Substrate-binding cavity (Å^3)
		W48-P156	N74-R152	Y209-R152		
EXP	Closed	3.95	5.22	7.84	44.4	909
ESM	Open	5.75	7.02	7.51	48.7	1046
AF2	Closed	5.13	6.03	7.47	46.3	812
AF3-1	Open	5.47	5.77	7.95	45.6	886
AF3-2	Closed	4.77	5.19	7.24	45.5	896
AF3-3	Closed	4.93	5.54	7.35	45.7	796
AF3-4	Open	5.64	6.28	8.33	46.9	961
AF3-5	Closed	5.16	5.67	7.89	45.3	818
RF-1	Open	6.47	8.70	6.24	52.1	1346
RF-2	Open	9.97	9.63	6.34	50.9	890
RF-3	Open	11.77	8.74	6.14	49.6	838
RF-4	Closed	10.75	9.28	6.86	52.3	1133
RF-5	Open	9.17	8.93	5.95	50.9	500

Table S2. Summary of substrate docking results for experimental and AI-predicted HviGH11.

Model	Pose	Docked substrate subsites	Docking Binding affinity (kcal/mol)	Glu97–substrate distance (Å) [1]	Glu207–substrate distance (Å) [1]
EXP	1	+2, +1, −1, −2, −3	−12.5	4.87	3.80
	2	−1, −2, −3		5.62	3.58
	3	+2, +1, −1, −2		5.79	3.24
ESM	1	+3, +2, +1, −1, −2, −3	−12.1	4.76	3.36
	2	N/A			
	3	N/A			
AF2	1	+2, +1, −1, −2, −3	−12.5	5.39	3.16
	2	N/A			
	3	−2, −3			
AF3-1	1	+3, +2, +1, −1, −2, −3	−12.8	5.19	3.66
	2	N/A			
	3	+2, +1, −1		5.93	5.74
RF-1	1	+1, −1, −2, −3	−11.0	4.95	3.48
	2	N/A			
	3	N/A			

N/A indicates poses in which the substrate is located outside the substrate-binding cleft. [1] The closest distances from the OE atoms of Glu207/Glu97 to the glycosidic oxygen between the xylose units at subsites +1 and −1.
